# Editorial: Traditional Chinese Medicine: Organ Vascular Injury - Volume II

**DOI:** 10.3389/fphys.2021.677858

**Published:** 2021-06-11

**Authors:** Jing-Yan Han, Gerald Meininger, Jin-Cai Luo, Qiao-Bing Huang

**Affiliations:** ^1^Department of Integration of Chinese and Western Medicine, Peking University Health Science Center, Beijing, China; ^2^Department of Medical Pharmacology and Physiology, Dalton Cardiovascular Research Center, University of Missouri-Columbia, Columbia, MO, United States; ^3^Beijing Key Laboratory of Cardiometabolic Molecular Medicine, Institute of Molecular Medicine, Peking University, Beijing, China; ^4^Guangdong Provincial Key Lab of Shock and Microcirculation, Department of Pathophysiology, School of Basic Medical Sciences, Southern Medical University, Guangzhou, China

**Keywords:** cardiovascular disease, inflammation, organ injury, hyperlipidemia, hyperglycemia, cerebrovascular, microcirculation

Hypertension, hyperlipidemia, diabetes, cardiovascular disease, cerebrovascular disease, kidney damage, chronic stress, inflammatory responses, and diseases caused by endotoxin, etc. are all major diseases that seriously threaten people's health and increase medical financial burdens worldwide. These diseases are characterized by an interacting network of abnormalities, resulting from genetic factors, unhealthy lifestyle, stress, immunological factors, environmental factors etc. It is increasingly important to recognize that these diseases are frequently conditions that involve multi-gene, multi-protein, and multi-cellular and tissue involvement. Thus, the pathways leading to and maintaining complex diseases are frequently multi-pathway. Currently, a host of single-component medicines are available to deal with these diseases, respectively, e.g., antihypertensive drugs for hypertension, lipid-lowering drugs for hyperlipidemia, and hypoglycemic agents for diabetes, which are effective. However, they a range of pathologically related manifestations linked to these conditions remain unresolved, including, for example, the reperfusion injury caused by recanalization after cardiac infarct and stroke, and the combination of microvascular and large vessel injuries caused by hypertension, hyperlipidemia, and diabetes.

Traditional Chinese Medicine (TCM) is a medical system with an extensive history spanning more than 2,000 years. Fundamental to TCM is an underlying philosophy of using a multi-target approach to complex disease. Over the long period of TCM evolution an abundance of clinical wisdom and effective compound preparations have been developed and accumulated that aid in the prevention and treatment of diseases related to vascular and organ damage. This has been of great advantage in China for combating complex diseases as in prevention and treatment of hypertension, hyperlipidemia, diabetes, cardiovascular, cerebrovascular diseases, chronic stress, and diseases caused by endotoxin. The TCM approach has also been of societal benefit in reducing the financial costs of medical treatments.

Professor G. A. Meininger and I organized a Research Topic for Frontiers in Physiology—Vascular Physiology, and received 76 manuscripts from 2017–January 5, 2018, of these 29 were accepted and published after peer review. In addition, Professors Meininger, Qiaobing Huang, Jincai Luo, and I organized an extension of This Research Topic for Frontiers in Physiology-Vascular Physiology to address the topic of “Traditional Chinese Medicine and Organ Vascular Injury,” and received 67 manuscripts from 2018 to 2020, among which 26 were accepted and published after peer review. This Research Topic also is cross-listed with the Ethnopharmacology section of Frontiers in Pharmacology, in which 10 were finally accepted and published after peer review. Here we compile these collected articles into an ebook to help scientists in the field learn and gain insight into TCM and its applications to complex diseases as well as TCM and organ vascular injury.

## The Effects and Mechanisms of TCM on Improving Treatment of Hypertension and Related Organ Injury

Among the papers included in this ebook, there are 4 papers regarding the mechanisms of TCM to improve treatment of hypertension. Xin et al. from the team of professors Xiao-Chun Xu and Xiang-Hong Jing demonstrated that electroacupuncture at PC6 for 30 min per day, for a period of 8 weeks, could inhibit the myocardial fibrosis in spontaneously hypertensive rats (SHRs), which might be mediated by downregulation of the activity of the AngII-TGF-β-CTGF/TNFα pathway, and upregulation of the diminished expression of MMP-9. Jiao et al. from the team of professors Jing-Yan Han and Zhi-Zhong Ma found that SHR rats manifested not only insufficient cerebral perfusion. This was accompanied by high expression of caveolin-1 in extracted proteins from brain tissues, low expression of tight junction proteins such as Claudin-5, Occludin, JAM-1, ZO-1, disarrangement and discontinuity of claudin-5 and ZO-1, albumin leakage from cerebral microvessels, Evans blue extravasation and increased cerebral edema. Enalapril plus nifedipine can lower blood pressure and improve cerebral perfusion, but have no effect on the cerebral microvascular effusion. YangXue QingNao Wan, a compound Chinese medicine for treating headaches and dizziness in China, can not only lower blood pressure and improve cerebral perfusion, but also inhibited the high expression of cerebral caveolin-1 and the low expression of tight junction proteins such as Claudin-5, Occludin, JAM-1, ZO-1, inhibited the disordered and discontinuous arrangement of Claudin-5 and ZO-1, and attenuated plasma albumin leakage from cerebral microvessels. There was also reduced Evans blue extravasation and cerebral edema, upregulation of ATPα and ATP5D, the two subunits of mitochondrial complex V, in extracted protein from brain tissue, elevation of the ratio of ATP/ADP and ATP/AMP, and inhibition of the damage of hippocampal neurons (Jiao et al.). Hao et al. from the team of professor Jing-Yan Han also demonstrated that Rhynchophylline, one of the main components of YangXue QingNao Wan, was capable of activating Src-PI3K/Akt-eNOS cascade and improving endothelium-dependent relaxation in the renal arteries from SHRs (Hao et al., 2017). Song et al. from the team of Professors Huayun Yu and Zhichun Wu demonstrated that Zi Shen Huo Luo formula could enhance the efficacy of angiotensin-converting enzyme inhibitors in left ventricular hypertrophy after hypertension by improving aldosterone activity and affecting mineralocorticoid receptor and Caveolin-1 colocalization and the downstream EGFR signaling pathway.

## The Effects and Mechanisms of TCM on Improving Treatment of Hyperlipidemia and Related Organs Injury

There are 4 papers regarding the improvement effects of TCM on hyperlipidemia and related cognitive dysfunction and arteriosclerosis. Gao et al. from the team of professors Yuan-Lu Cui and Chun-Quan Yu demonstrated that Dan-Lou prescription effectively attenuated macrophage foam cell formation induced by ox-LDL via the TLR4/NF-κB and PPARγ signaling pathways (Gao et al., 2018). Yi et al. from the team of professors Fei Ye and Xiaolin Zhang demonstrated that Rho, a extract from *Rhodiola crenulate*, could ameliorate hepatic steatosis in high-fat diet (HFD)-induced obesity mice, KKAy mice, and HFD combined with tetracycline stimulated Model-T mice, respectively. The mechanism was related to enhancing insulin sensitivity, suppressing fatty acid uptake and inhibiting *de novo* lipogenesis in liver (Yi et al., 2018). Gu et al. from the team of professors Jing-Yan Han and Zhi-Zhong Ma demonstrated that YangXue Qing Nao Wan and Silibinin Capsules could improve cognitive impairment of aged LDLR (+/–) golden Syrian hamsters, which was associated with attenuated albumin leakage from middle cerebral artery and neuronal damage in hippocampus, concomitant with an increase in cerebral blood flow, a decrease of perivascular edema and an up-regulated expression of claudin-5, occludin and ZO-1 (Gu et al., 2018). Deng et al. from the team of professor Jing-Yan Han also demonstrated that combination of Cardiotonic Pills, a compound Chinese medicine for coronary heart disease and angina pectoris, and recombinant human prourokinase, a thrombolytic drug, effectively attenuated atherosclerosis plaque in LDLR^−/−^mice plus high fat diet, which is associated with normalizing the lipid metabolism in the liver and aorta, reducing phagocytosis of receptor-mediated modified-LDL uptake and inhibiting systemic inflammation (Deng et al., 2019).

## The Effects and Mechanisms of TCM on Improving Treatment of Hyperglycemia and Related Organs Injury

We have included 5 papers reporting the effect of TCM on diabetes, especially the vascular comorbidity and related organ injury. Li et al. from the team of professors Qiaobing Huang and Xiaohua Gao showed that Src plays a pivotal role in advanced glycation end products-promoted HUVECs angiogenesis by phosphorylating ERK, and very likely through RAGE-Src-ERK pathway (Li et al., 2018b). Zhu et al. from the team of professors Ximing Liu and Xiaobo Sun found that Dai-Zong-Fang, a traditional Chinese herbal formula containing berberine, naringin, and other components, exhibits a prominent effect in improving insulin sensitivity, hepatic steatosis, and skeletal muscle energy metabolism in db/db mice (Zhu et al., 2018a). Cheng et al. from the team of professors Lin Yao and YongJun Chen reported that HuangqiGuizhiWuwu Decoction improved streptozocin-induced vascular dysfunction by targeting endothelial arginase 1. Chen et al. from the team of professors Jianxun Liu and Junguo Ren discovered the therapeutic potential of Tang Wang Ming Mu Granule, a compound Chinese medicine, for prevention of diabetic retinopathy in Type 2 diabetes rats, which may be attributable to anti-inflammation, anti-oxidation, upregulation of SOCS3 expression, and inhibition of the JAK/STAT/VEGF signaling pathway (Chen et al., 2017). Li et al. from the team of professors Jing-Yan Han and Xue-Mei Wang reported that Bushen Huoxue (BSHX), a compound Chinese medicine, significantly ameliorated the type 2 diabetes mellitus related insults in diabetic KKAy mice, including the increased blood glucose, the impaired spatial memory, decreased cerebral blood flow, occurrence of albumin leakage, leukocyte adhesion and opening capillary rarefaction. Meanwhile, the downregulation of the tight junction proteins claudin-5, occludin, zonula occluden-1 and JAM-1 between endothelial cells, the amyloid-β accumulation in hippocampus, increased advanced glycation end-products and RAGE, and expression of RhoA/ROCK/moesin signaling pathway and phosphorylation of Src kinase were significantly protected by BSHX treatment. These results indicate that the protective effect of BSHX on type 2 diabetes mellitus-induced cognitive impairment involves regulation of RhoA/ROCK1/moesin signaling pathway and phosphorylation of Src kinase (Li et al., 2018c).

## The Effects and Mechanisms of TCM on Improving Treatment of Cardiac Injury, Myocardial Fibrosis and Heart Failure

We have included 22 papers regarding the improvement effects of TCM on cardiovascular injury, including 1 paper investigating the role of TCM in myocardial injury caused by homocysteine, 6 papers about the improvement of ischemic myocardial injury by TCM, 5 papers about the improvement of myocardial ischemia-reperfusion injury by TCM, 4 papers reporting the protective effect of TCM on heart failure, 3 papers regarding the systematic biological characteristics of cardiovascular disease with different syndromes of TCM, 3 papers regarding the effect of TCM on coronary heart disease in clinic.

### The Effects of TCM on Improving Treatment of Homocysteine-Induced Cardiac Injury

The work of Fan et al. from the team of professors Bao-liang Sun and Xiao-yan Fu showed the suppressed effect of astaxanthin on homocysteine-induced cardiotoxicity *in vitro* and *in vivo* by inhibiting mitochondrial dysfunction and oxidative damage (Fan et al., 2017).

### The Effects and Mechanisms of TCM on Improving Treatment of Ischemic Myocardial Injury

Zhang et al. from the team of professors Pengfei Tu and Yong Wang revealed the anti-apoptotic effects of Baoyuan Decoction both *in vivo* and *in vitro*, is mediated by regulation of the P38 mitogen-activated protein kinase-αB-crystallin signaling pathway (Zhang et al., 2018b). Cheng et al. from the team of professor Mingjing Zhao reported that 1 day after left anterior descending coronary artery ligation, continuous administration of Qiliqiangxin Capsules (QLQX) by intragastric administration for 8 weeks can reduce the area of myocardial infarction, improve cardiac function and myocardial blood flow. The role of QLQX is attributable to inhibition of cardiac glycolysis and promotion of glucose oxidation and fatty acid metabolism (Cheng et al.). Chen et al. from the team of professors Xiumei Gao and Guanwei Fan revealed that Danhong Injection combined with mesenchymal stem cells intervention can significantly improve cardiac function and inhibit ventricular remodeling in murine myocardial infarction model. This benefit may be due to the regulation of the SDF-1/CXCR4 axis and promote angiogenesis (Chen et al., 2018). Sun et al. from the team of professors Xiaohui Ma and Feng Yu demonstrated the protective effect of compound danshen dripping pills on isoproterenol-induced myocardial ischemia in rat and the acute myocardial infarction rat induced by ligation of the left anterior descending (LAD) coronary artery, whereas the circulating microRNA-1 was only ameliorated in the LAD rat model (Sun et al., 2018b). Cui et al. from the team of professor Jing-Yan Han found that treatment with QiShenYiQi Pills (QSYQ) significantly inhibited ischemia-induced rat myocardial injury, as demonstrated by the improvement of myocardial morphology and heart function and decline in cardiac cTnI release. QSYQ also relieved myocardial energy disorders manifested by the increase in ATP production, ATP 5D protein expression, and ATP synthase activity. AS-IV and Rb1, but not Rg1, R1, and DLA, had similar effect as QSYQ in regulation of energy metabolism (Cui et al., 2018). Fu et al. from the team of professor Qi-Tao Zhao demonstrated that Trichosanthes pericarpium aqueous extract displays protective effect on acute myocardial infarction by promoting the mobilization of endothelial progenitor cells and up-regulating the expression level of VEGF, eNOS, NO, and MMP-9 in myocardium and their plasma content in acute myocardial ischemic rats (Fu et al., 2018).

### The Effects and Mechanisms of TCM on Improving Treatment of Myocardial Ischemia and Reperfusion Injury

**Figure 1:**
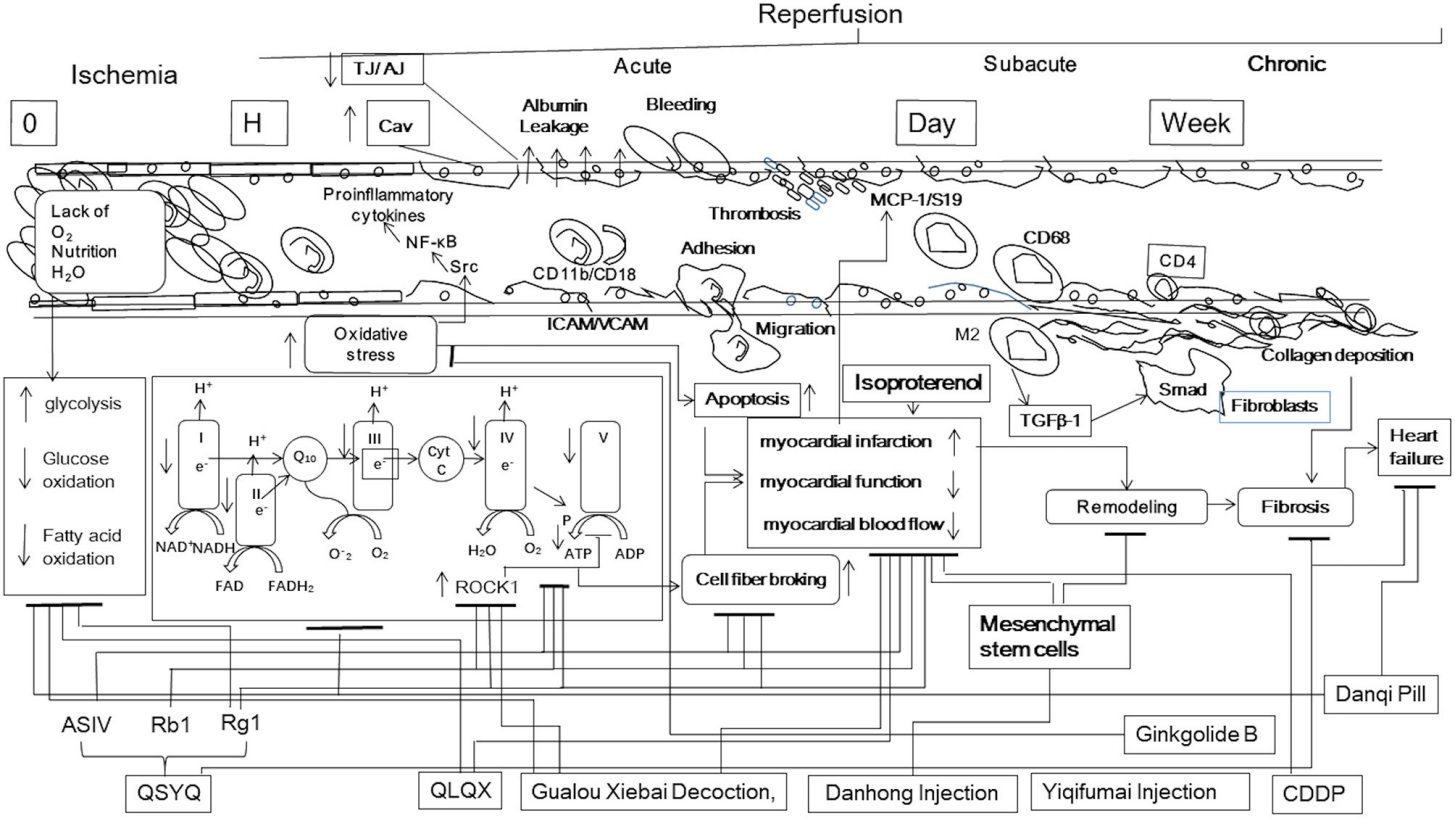
I/R-induced microcirculatory disturbance and tissue injury stepwise progress involving the ischemia, acute, subacute, and chronic phases after reperfusion. Some TCM showing attenuation effect on I/R-induced microcirculatory dysfunction and tissue injury at various phases. I, mitochondrial complex I; II, mitochondrial complex II; III, mitochondrial complex III; IV, mitochondrial complex IV; V, mitochondrial complex V; ADP, adenosine diphosphate; AJ, adhesion junction; ASIV, astragaloside IV; ATP, adenosine triphosphate; Cav, caveolae; CDDP, compound danshen dripping pills; Cyt C, cytochrome c; FAD, flavin adenine dinucleotide; ICAM, intercellular adhesion molecule; M2, M2 macrophages; MCP-1, monocyte chemotactic protein 1; NAD, nicotinamide adenine dinucleotide; NF-κB, nuclear factor kappa-B; Q10, Coenzyme Q10; QLQX, QiLiQiangXin capsule; QSYQ, QiShenYiQi; Rb1, Ginsenoside Rb1; Rg1, Ginsenoside Rg1; ROCK, Rho-associated kinase; S19, ribosomal protein S19; TGF-β1, transforming growth factor-β1; TJ, tight junction; VCAM, vascular cell adhesion molecule. ↑, increase; ↓, decrease; ⊥, inhibition.

Yan et al. from the team of professors Jing-Yan Han and Xin-Sheng Yao revealed that Gualou Xiebai Decoction, a compound Chinese medicine, significantly protected the myocardium from I/R injury in hyperlipidemia rat, as shown by the decreased level of CK, CK-MB, LDH, cTnI, cTnT, and IL-6, improved cardiac function, and mitigated myocardium damage, possibly via regulating energy metabolism involving inactivation of RhoA/ROCK signaling pathway (Yan et al., 2018). Zuo et al. from the team of professors Hua Zhou and Pei Luo demonstrated that an acid polysaccharides fraction of *Panax ginseng* exerted a protective effect in H9c2 cardiomyocytes underwent hypoxia/reoxygenation on mitochondrial pathway apoptosis via elevating the expressions of glucocorticoid receptor and estrogen receptor and activating eNOS increasing NO production (Zuo et al., 2018). Li et al. from the team of professors Jing-Yan Han and Li Huang demonstrated that ginsenoside Rg1, a major ingredient of *Radix ginseng*, protected heart from I/R-induced myocardial impairment, as shown by the decrease of myocardial infarction size, the increase in myocardial blood flow, and amelioration of cardiomyocyte structure and function. The cardiac protection afforded by ginsenoside Rg1 may be attributed to its ability to adjust energy metabolism via regulating the expression of energy metabolism related proteins (HIF1, ENOα, ALDO, ECH1), increasing the activity of mitochondria respiratory complexes and the expression of ATP5D, all of which may at least partially be mediated through its binding to RhoA to inactivate the RhoA/ROCK pathway (Li et al., 2018a). Zheng et al. from the team of professors Yan Wang and Guo-Qing Zheng conducted a preclinical systematic review to evaluate the effectiveness and the mechanisms of Astragaloside IV for myocardial I/R injury, and the findings indicate that Astragaloside IV exerted potential cardioprotective function in acute myocardial I/R injury largely through promoting angiogenesis, improvement of the circulation, antioxidant, anti-apoptosis, and anti-inflammatory (Zheng et al., 2018). Zhu et al. from the team of professors Yan Wang and Guo-Qing Zheng performed a systematic review over the available preclinical evidence and possible mechanisms of Ginkgolide B in treating myocardial I/R injury, finding that the possible mechanisms via which Ginkgolide B exerts cardioprotective effects are mainly associated with anti-oxidation, anti-inflammation, anti-apoptosis, and improvement of energy metabolism (Figure 1).

### The Effects and Mechanisms of TCM on Improving Treatment of Heart Failure

Zhang et al. from the team of professors Yong Wang, Wei Wang, and Chun Li demonstrated that Danqi Pill could improve myocardial glucose metabolism, mitochondrial oxidative phosphorylation and biogenesis by regulating HIF-1α/PGC-1α signaling pathway in heart failure post-acute myocardial infarction. Mao et al. from the team of professor Minzhou Zhang reported that Tongguan Capsule improves cardiac remodeling in a murine model of myocardial infarction by preventing cardiomyocyte inflammation and apoptosis while enhancing autophagy through Sirt1 activation (Mao et al., 2018). Ruan et al. from the team of professor Liyue Wang showed that treatment with QiShenYiQi Dripping Pills could protect against transverse aortic constriction-induced cardiac dysfunction, disrupted myocardial fiber structure and heart failure. The mechanism may be associated with the improvement in impaired cardiac angiogenesis (Ruan et al., 2018). Zhao et al. from the team of professors Yi Wang and Guanwei Fan found that Yiqifumai Injection could alleviate cardiac hypertrophy and apoptosis, and reduce cardiac fibrosis and heart failure caused by ligation of the left anterior descending coronary artery in rats, which is associated with the upregulation of MiR-21-3p and miR-542-3p (Zhao et al., 2018).

### Systematic Biological Characteristics of Different Syndromes of TCM in Cardiovascular Disease

Zhao et al. from the team of professor Dong-Sheng Wang proposed a metabolomics method based on ^1^H-NMR and random forest models to elucidate the underlying biological basis of blood stasis syndrome in coronary heart disease (CHD). Choline, β-glucose, α-glucose, and tyrosine were considered as potential biomarkers of CHD-blood stasis syndrome (Zhao et al.). Zhu et al. from the team of professor Wuxun Du investigated the metabolic profiles of plasma samples from myocardial infarction (MI) patients with phlegm stasis syndrome to identify potential disease biomarkers. Significant difference in the plasma levels of the following 10 metabolites was observed in the MI patients compared with the controls: phosphatidylserine, C16-sphingosine, N-methyl arachidonic amide, N-(2-methoxyethyl) arachidonic amide, linoleamidoglycerophosphate choline, lysoPC (C18:2), lyso-PC (C16:0), lyso-PC (C18:1), arachidonic acid, and linoleic acid (Zhu et al., 2018b). Xu et al. from the team of professors Xue-Zhong Zhou and Jing-Qing Hu proposed a network medicine-based approach to identify the characteristic of proteins in ischemic heart disease patients with phlegm-stasis syndrome, which could be used for interpreting the pharmacological mechanisms of well-established Chinese herbal formulas (e.g., Tao Hong Si Wu Decoction, Dan Shen Yin Decoction, Hunag Lian Wen Dan Decoction, and Gua Lou Xie Bai Ban Xia Decoction) (Xu et al., 2018).

### The Effects of TCM on Coronary Heart Disease Patients

Tao et al. from the team of professor Xiuhua Liu investigated the metabolic profiles of the serum in CHD patients after treatment of Taohong Siwu Decoction (THSWD) using non-targeted ultra-performance liquid chromatography with tandem mass spectrometry-base metabolomics. The results indicated that THSWD promoted energy generation by upregulating fatty acid metabolism and downregulating tricarboxylic acid cycle and pentose phosphate pathway. THSWD also downregulated glycerophospholipid metabolism and arachidonic acid metabolism and involved amino acid metabolism. They showed that small molecular metabolites such as glycerophosphocholine, 8,9-DiHETrE, 5′-MTA, hippurate, indoxyl sulfate, and 3-UPA may be the potential targets of THSWD for anticoagulation and lipid reduction, and the material foundation of the “blood” to promote blood circulation in CHD treatment (Tao et al.). Following a systematical review, Yang et al. from the team of professor Ya-Hong Wang claims an effect of Tai Chi on cardiorespiratory fitness and coronary heart disease rehabilitation (Yang et al., 2018b). Based on a retrospective study on the National Health Insurance Data, Yang et al. from the team of professors Sheng Han and Hao Hu evaluated the clinical efficiency and economics of salvianolate injection for patients with CHD in comparison to Danhong injection and alprostadil injection. The result indicated the advantage of Salvianolate injection in reducing hospitalization duration for inpatients with CHD (Yang et al.).

## The Effects and Mechanisms of TCM on Cerebrovascular Diseases

In cerebrovascular disease, 8 articles are included, of which 3 are studies on improving effects of TCM on cerebral ischemic injury and 5 on cerebral ischemia and reperfusion injury.

### The Effects of TCM on Improving Treatment of Cerebral Ischemic Injury

Zhang et al. from the team of professors Yan Wang, Guo-Qing Zheng, and Jie Liu using a mouse cerebral ischemic model evaluated the effect of borneol, finding that mesenchymal stem cells (MSCs) plus borneol was more effective in attenuation of neurological deficits, infarct volume, cell death, and neurogenesis than MSCs alone (Zhang et al., 2018a). Li et al. from the team of professor Hui Zhao reported that Xiaoshuan enteric-coated capsule along with enriched environment exerted synergistic effects on alleviating atrophy and encouraging axonal reorganization partially by promoting oligodendrogenesis and overcoming intrinsic growth-inhibitory signaling, thereby facilitating higher cognitive recovery. Xue et al. from the team of professors Zhuoya Ma and Shuguang Liu based on a review and meta-analysis suggested that the combination of conventional treatments and Ginkgo leaf extract and dipyridamole injection is safe and more effective in treating ischemic stroke than conventional treatments alone.

### The Effects of TCM on Improving Treatment of Cerebral Ischemia and Reperfusion Injury

Zhao et al. from the team of professors Jiehong Yang and Haitong Wan showed that many influencing factors could affect the compatibility of components contained in two formulas (CG4 and CG6), such as the metabolism by CYP450 enzymes, plasma protein binding rates, and effects related to the blood-brain barrier. This study provided new insights for choosing appropriate dosages of active components of TCM to aid in prevention and treatment of cerebral ischemic diseases (Zhao et al.). Yang et al. from the team of professor Guo-Qing Zheng reported that the herbal compatibility of Ginseng and Rhubarb synergistically exerted neuroprotective function during acute cerebral I/R injury, mainly through reducing the expression of connexin-43 and aquaporin-4. Fu et al. from the team of professor Guo-Qing Zheng investigated the neuroprotective effects of Sanhua Decoction, a classic Chinese herbal prescription, on cerebral I/R injury in rat model, showing that Sanhua Decoction exerted neuroprotection probably by regulating p-tau level and promoting the proliferation, migration, and differentiation of endogenous neural stem cells, accompanying with neurobehavioral recovery. Wang et al. from the team of professors Yong Jiang and Kewu Zeng demonstrated that as the main active components of *Cistanche deserticola*, total Glycosides have porential to protect against I/R-induced cerebral injury, and the protection was mainly via the Nrf-2/Keap-1 pathway. Chen et al. from the team of professor Jiangang Shen discuss the important roles of myeloperoxidase (MPO) in mediating oxidative stress and neuroinflammation during cerebral I/R injury and summarize the active compounds from medicinal herbs with potential as MPO inhibitors for anti-oxidative stress and anti-inflammation to attenuate cerebral I/R injury, and as adjunct therapeutic agents for extending the window of thrombolytic treatment.

## The Effects and Mechanisms of TCM on Improving Treatment of Kidney Injury

Two research papers and 1 review article are included with respect to the improvement of kidney injury by TCM. An et al. from the team of professor Lin Yao and Yongjun Chen demonstrated that the protective effect of Xiao-Shen-Formula on kidney injury might be related with vascular prevention, anti-inflammation and anti-oxidation through intervening with multi-targets including glomerular endothelial arginase-heparanase signaling pathway in diabetic nephropathy model (An et al., 2018). Zhou et al. from the team of professor Jing-Yan Han demonstrated that pretreatment with QiShenYiQi Pills (QSYQ), a compound Chinese medicine with potential of tonifying Qi and activating blood, significantly attenuated the cisplatin induced insults, including the increase in plasma urea and creatinine, histological damage, and the renal microcirculation disturbance. These effects were ascribed to its ability to upregulate mitochondrial respiratory chain Complex I, II, and IV thus containing the production of peroxides and apoptosis, and elevate the expression of the complex V increasing ATP yield (Zhou et al., 2017). Lv et al. from the team of professor Richard J. Roman in a review article concerning oxidative stress and renal fibrosis revealed that a spectrum of TCM were reported effective in attenuation of renal injury, including compound Chinese medicines Liu-Wei-Di-Huang-Wan, Ba-Wei-Di-Huang-Wan, Fufang Xue Shuan Tong, Hu-Lu-Ba-Wan, Chinese material medica Dan-Shen, Xuan-Shen, Huang-Qi, San-Qi, Chuan-Xiong, Shan-Zha, Ge-gen, bioactive ingredients magnesium lithospermate B, caffeic acid, Icariin, Berberine, Curcumin, Paeoniflorin, Ferulate, Triptolide, Total flavonoids, Taxol, etc. (Lv et al., 2018).

## The Effects and Mechanisms of TCM on Organ Injury Caused by Chronic Stress

Two papers are included that addressed the effect of TCM on organ injury caused by chronic stress. Sun et al. from the team of professor Jing-Yan Han demonstrated the potential of Xiao-Yao-San, a classic Chinese medicine formula, to ameliorate follicle development abnormalities in a polycystic ovary syndrome rat model caused by chronic stress. The study revealed that the beneficial role of Xiao-Yao-San was correlated with the inhibition of elevated beta hydroxylase in locus coeruleus, noradrenaline release, β2R expression and apoptosis and autophagy of granulosa cells (Sun et al., 2017). Chen et al. from the team of professor Changjiang Hu reported that Huangsiyujin and its processed products may alleviate pain by regulating the release of 5-hydroxytryptamine, increasing the content of β-endorphin and inhibiting the expression of c-fos in a rat model of Qi stagnation and blood stasis.

## Ameliorating Effects of TCM on Microcirculation Disorders and Organ Injury Caused by Endotoxin

Three papers are included that are devoted to identify effects of TCM on microcirculation disorder and organ injury caused by endotoxin. Wang et al. from the team of professor Jing-Yan Han demonstrated that post-treatment with Qing-Ying-Tang (QYT), a classic compound Chinese medicine, significantly ameliorated LPS-induced leukocyte adhesion to microvascular wall, albumin leakage from cerebral venules and brain tissue edema. The compound also attenuated the increase of MCP-1, MIP-1α, IL-1α, IL-6, and VCAM-1 in brain tissue and the activation of NF-κB and expression of MMP-9 in brain. QYT ameliorated the downregulation of claudin-5, occludin, JAM-1, ZO-1, collagen IV as well as the expression and phosphorylation of VE-cadherin in mouse brain. QYT protected cerebral microvascular barrier from disruption after LPS involving both transcellular pathway and paracellular pathway (Wang et al.). Sun et al. from the team of professors Jing-Yan Han and Xian Wang demonstrated that schisandrin (Sch), an ingredient of *Schisandra chinensis*, alleviated leukocyte adhesion to pulmonary venules and infiltration into lung tissue after LPS stimulation, which is attributable to inhibition of the increase in the expression of TLR-4, phosphorylation of I-κB, nuclear translocation of NF-κB, the expression of leukocyte adhesion molecules CD11b/CD18 and endothelial adhesion molecules ICAM-1 and VCAM-1. Sch ameliorated the downregulation of claudin-5, occludin, JAM-1, ZO-1, and lung endothelium and epithelium injury, attributable to the regulation of Akt/FoxO1 signaling pathway (Sun et al., 2018a). Wang et al. from the team of professors Chun-Yu Niu and Zi-Gang Zhao reported that resveratrol treatment partly reduced the whole blood viscosity and regional blood flow, and increase white blood cell content in peripheral blood following the LPS challenge, suggesting a favorable role in expanding the quasi-sympathetic effects of LPS in blood viscosity at early stages (Wang et al., 2018).

## Multi-Component Pharmacological Effects of Compound Chinese Medicine

There are 4 papers regarding the multi-link pharmacological effects of compound Chinese medicine. Gong et al. from the team of professors Tina TX Dong and Kelvin Chan reviewed the chemical compositions and pharmacological effects of “Snow lotus” in treating various disorders. Yang et al. from the team of professors Ping Li and Xuezhong Zhou using network pharmacological methods revealed that G-protein coupled receptor signaling pathway and cellular protein metabolic process are the key pathways for LianXia NingXin formula. This formula was used to treat coronary heart disease phenotypes with corticotropin releasing hormone and natriuretic peptide precursor A being the two key drug targets. Further evidences from Chinese herb pharmacological databases indicate that *Pinellia ternata* (Banxia) has relatively strong adjustive effect on the two key targets (Yang et al., 2018a). Liu et al. from the team of professors Xuan Liu, Ben He, and Xianting Ding proposed that feedback system control optimization technique could be used in optimization of anti-platelet drug combinations and might be helpful in designing personal anti-platelet therapy strategy. Furthermore, feedback system control analysis could also identify interactions between different drugs which might provide useful information for research of signal cascades in platelet (Liu et al., 2018). Zhang et al. from the team of professors Xuefeng Xiao and Wuxun Du provided a strategy for understanding the mechanism where by Qiliqiangxin capsule (QLQX) protected against chronic heart failure, which included pharmacokinetics study, network pharmacology, and experimental validation. A total of 29 ingredients were determined by pharmacokinetics study, instead of herb databases, and used for network pharmacology analysis. Through experimental validation of the hub targets (VEGFA, IL-6, p-STAT3, and p-JAK2), the JAK/STAT signaling pathway were identified as the mechanism by which QLQX attenuated inflammatory process in chronic heart failure (Zhang et al.).

## Author Contributions

J-YH and GM have contributed to the writing of this editorial. All authors contributed to the article and approved the submitted version.

## Conflict of Interest

The authors declare that the research was conducted in the absence of any commercial or financial relationships that could be construed as a potential conflict of interest.
